# Valid consent in the acute hospital setting: perspectives of nursing and medical professionals from a survey-based study

**DOI:** 10.1007/s11845-025-03952-1

**Published:** 2025-05-02

**Authors:** Charmaine Zahra, Motheo Kobua, Živa Kovic, Mary Fogarty, Catherine Buckley, Jane Murphy, Julie Walshe, Paul Zambra, Declan Byrne, Una Geary, Marie E. Ward

**Affiliations:** 1 Quality and Safety Improvement Directorate, St James’s Hospital, Dublin 8, Ireland; 2 School of Medicine, Trinity College Dublin, Dublin 2, Ireland; 3 Trinity St James Cancer Institute (TSJCI), St James’s Hospital, Dublin 8, Ireland; 4 Surgery, Anaesthetics, Critical Care, St James’s Hospital, Dublin 8, Ireland; 5 School of Psychology, Trinity College Dublin, Dublin 2, Ireland; 6 St James’s Hospital, Dublin 8, Ireland; 7 Centre for Innovative Human Systems, School of Psychology, Trinity College, Dublin 2, Ireland

**Keywords:** Acute hospital, Capacity, Informed consent, Valid consent, Voluntary consent

## Abstract

**Background:**

In healthcare, consent refers to the act of granting permission or agreement for treatment and care, investigation, receiving or utilising a service, or participating in research or teaching. Consent should be an ongoing process that involves clear communication about the proposed intervention, including its nature, benefits, and potential risks.

**Aim:**

This survey-based study gathered experiences from junior doctors and nurses in a large acute teaching hospital about current consent practices and suggestions for improvement.

**Method:**

Two surveys were developed and distributed to junior doctors in 2022 and nurses in 2023.

**Results:**

The response rate for junior doctors (*n* = 58) was 21% (interns) and 57% (senior house officers) and 10% of the total nursing population responded (*n* = 184). Descriptive statistics and content analysis were used to analyse the results. Both junior doctors and nursing professionals believed there were areas for improvement in terms of consent processes and practices including in relation to better information for patients and more education and training for healthcare professionals.

**Conclusions:**

The process of informed consent is central in the planning and provision of safe, effective person-centred healthcare as it encompasses healthcare professionals and patients communicating about and together deciding on and agreeing to medical interventions. This survey-based study looked at the experiences, attitudes, and perceived needs of junior doctors and nursing professionals in relation to the informed consent process in clinical practice at an acute hospital and informed the development of recommendations for improvement.

## Introduction

In healthcare, consent refers to the act of granting permission or agreement for treatment and care, investigation, receiving or utilising a service, or participating in research or teaching [1]. Consent should be an ongoing process that involves clear communication about the proposed intervention, including its nature, benefits, and potential risks. Obtaining consent is essential; it is also legally and professionally required as individuals have a legal and ethical right to make informed decisions regarding their bodies [1, 2].

For consent to be ethical and legally valid, the patient must be adequately informed to make the decision, have the capacity to make their own decision, and make the decision voluntarily [1, 3, 4]. The Assisted Decision-Making (Capacity) Act (ADMCA), which was enacted in Ireland in 2023, sets out among other provisions, that a patient’s capacity to make decisions must be assessed on a decision-by-decision basis [5]. A functional test must be applied to determine capacity for making each decision which involves assessing that the patient has understood the information relevant to the decision, has retained that information long enough to make a voluntary choice, has demonstrated that they can use or weigh that information as part of the process of making the decision, and can communicate their decision [5].

Consent can be either explicitly given (in written form or verbally) or can be implied through cooperation (for example, a patient holding out their arm to have their blood pressure taken). Regardless of the method used for confirming the patient’s consent, it is recommended that healthcare professionals (HCPs) should document what was discussed and agreed upon. Signed consent is recommended for invasive or increased risk procedures [1, 3]. Revisiting consent decisions is appropriate in the case of a change in the patient’s condition, or substantial time has passed since the initial informed consent was obtained. Obtaining consent well in advance of major procedures and interventions is good practice to avoid the patient having to make rushed decisions [1].

Obtaining valid consent may require multiple discussions between the patient and the HCP, especially in the case of major procedures and interventions. Patients should be informed about any changes in their clinical condition impacting the consent process and any changes to the proposed procedure/intervention for which they consented. HCPs should ensure that the patient consents to any changes in the planned procedure and has no further questions or concerns. This requires an ongoing meaningful and open dialogue in a way that empowers patients to fully understand the potential risks, benefits, and consequences of each option and also to decline any treatment [3, 6, 7].

Good consent practices underpin shared decision-making (SDM). SDM is a collaborative process that involves a patient and their HCP working together to reach a joint decision about care. SDM involves choosing tests and treatments based both on evidence and on the patient’s individual preferences, beliefs, and values. It means making sure the patient understands the risks, benefits, and possible consequences of different options through discussion and information sharing [8]. SDM relies on two sources of expertise: the HCP as an expert on the effectiveness, probable benefits and potential harms of treatment options and the patient as an expert on themselves, their social circumstances, attitudes to illness and risk, values, and preferences [8]. Good consent practices should provide opportunities for this sharing of knowledge and expertise.

Various challenges and barriers have been identified in the literature regarding obtaining valid consent and SDM in medical practice which include but are not limited to: clinician related factors, such as the lack of clear and accessible communication, the lack of awareness of power dynamics, limited knowledge and skills for SDM, the lack of cultural awareness, and the lack of legal compliance; patient related factors such as language barriers, past negative experiences; organisational and system related factors include the lack of appropriate environments for consent discussion to take place and the lack of guidelines [6–12].

Consultants have described their challenges to obtaining valid consent including the vulnerability of patients in the medical setting, which can lead to a potential imbalance of power between consultants and patients [10], and patients wanting ‘either to sign the consent form without acknowledging information, or deferring the decision to the doctor’ [10 p.757], time for consent conversations and inadequate information sharing, and ineffective risk communication [10].

Junior doctors in Ireland are doctors who have graduated from medical school in the last year (interns) and 2 to 3 years (senior house officers (SHOs)). They play a large role in consent practices in hospital settings. Junior doctors may have gaps in their knowledge of interventions and their risks and benefits [12], have consented to procedures they are not familiar with [13], and require further training on consent law [14, 15].

Nurses also play a large role in consent practices in hospitals [16–18]. They face challenges due to a lack of clarity about their roles and responsibilities in relation to consent including inadequate official policies, procedures, protocols, and guidelines (PPPGs) [19, 20], excessive workloads, high number of patients, believing that consent is required for interventional procedures only [21], or the patient is compliant and therefore consent is implied [22, 23].

Patients play an important role in the consent process and their desires in relation to consent practices have been studied [24–26]. Challenges patients face are feeling that the consent process was more about obtaining a signature rather than having a meaningful conversation about a planned course of action [24], being left with outstanding questions about their procedures [25] and a desire to receive more written information on their treatment [25, 26]. Patient anxiety and fear of mortality may contribute to a lack of desire to be fully informed during the consent discussion [25]. What patients do wish to have more information about is the potential impact of the treatment on their daily lives [26], and there is a recognition among HCPs that patient safety is directly linked to patient comprehension of the informed consent process [27].

The aim of this survey-based study was to gather insights from junior doctors and nursing professionals regarding their experiences of consent conversations and practices and their recommendations for how to improve consent practices.

## Materials and methods

### Study context and ethics

This study was conducted at a large acute teaching hospital. The hospital is a tertiary referral hospital and has over 1000 patient beds and 4800 healthcare professionals. At the time of the survey study, there were 68 interns, 80 SHOs, and 1927 nursing professionals working at the hospital. Participants were emailed participant information leaflets (PILs) on the study and invited to participate using an electronic survey link. Participation was on a voluntary basis. No personal data was collected from participants. As both surveys were completed anonymously and voluntarily, participants’ informed consent was implied through the completion of the survey. All participants in the study were adults and assumed to be in a position to give their own informed consent. The need for ethics approval was waived with the approval of the hospital’s institutional review board (Ref: SJH R&I 7487). The study was carried out in accordance with the hospitals guidelines and regulations for undertaking service evaluation and improvement projects.

### Survey development

In this survey-based study due to the different roles that junior doctors and nursing professionals have in consent practices, two different surveys (see Appendices 1 and 2) were co-designed, for each group. The surveys have a set of 23 and 16 questions, respectively, consisting of yes/no answer questions, multiple-choice questions, and short answer options.

The study project team, consisting of the director for quality and safety improvement (UG), effective care and assurance quality manager (MF), health system learning and research (MEW), business manager (PZ), and clinical lead (DB), supported the co-design of the surveys. Co-design in healthcare involves the equal partnership of those who work within the system, those who use the system, and those who design interventions for the system, with a shared goal of achieving better outcomes or more efficient processes [28].

Two medical students (MK & ŽK), following a review of the literature, also supported the co-design of the survey for the interns and SHOs. The questions in Section A of the junior doctor survey were primarily taken from Heaney et al. [12]. Section B questions were situational statements derived from the national consent policy, as well as open-ended questions to explore what the barriers to good consenting practices were and gather suggestions for improvement. Two representatives from nursing professionals (CB & JM) in the hospital also supported the co-design of the survey for nursing professionals based on the survey for junior doctors but with the following changes; the survey was shortened, and a focus was put on how nurses currently deal with challenging situations, for example, in relation to times when patients decline to give their consent. The open-ended qualitative questions were the same across both surveys.

Both surveys were piloted to assess their usability and face validity. The junior doctors survey was piloted with four interns and SHOs, whilst the nursing survey was piloted initially by five nursing professionals and later by ten nursing professionals as part of the hospital nursing research ethics group review. All suggested changes from these reviews were implemented.

### Survey administration

The survey link and the PIL were sent out electronically, via the hospital medical education facility, to all of the 80 SHOs and 68 interns who were working in the hospital at the time. The survey link and PIL were sent out electronically, via nursing management, to all 1927 nursing professionals working in the hospital at the time. However, it must be noted that not all nursing professionals have regular access to email. To prevent unauthorised access and multiple participation, the survey link was distributed through the hospital's internal email system. Junior doctors were surveyed using Qualtrics XM Platform TM, while SurveyMonkey® was used for nursing professionals. In the nature of co-design, the different professional groups could chose survey platforms that they had access to and were familiar with, hence two different platforms. Surveys were left open for a period of 4 weeks.

### Survey data analysis

Descriptive statistical techniques, supported by Microsoft Excel, were employed, after data cleaning, to analyse the survey responses. Percentage responses were calculated out of the total number of responses to each question as indicated in each table. Similarly, for questions ‘tick all that apply’, the percentage of the total responses were calculated.

Free text responses were analysed using content analysis with the help of Microsoft Excel, following published methods [29, 30] to create a classification of themes that recur across the data set. Ten per cent of the nursing responses were double coded by two of the authors (CZ & MEW), and 10% of the doctors’ responses were double coded by two authors (JW & MEW).

The Checklist for Reporting Of Survey Studies (CROSS) quality appraisal tool for carrying out web-based and non-web-based surveys [31] was used and can be found in the Supplementary files.

## Results

### Survey response rates

A total of 58 junior doctors responded, and 1 survey response was invalid and discarded. Out of 57 valid responses, 39 (70%) were interns, and 17 (30%) were SHOs (1 did not specify), giving response rates of 57% for interns and 21% for SHOs. A total of 184 responses were received from nursing professionals. Not all nursing professionals may have received the invitation to complete the survey as not all nursing professionals have access to email. We therefore estimate our response rate as 10% of the total nursing population at the time of the study (1927 nursing professionals).

### Survey results

The survey results are presented here for both junior doctors and nursing professionals under the following headings:

- Role and responsibilities of professionals in the consent process
- Information and knowledge
- Education and training
- Supports for professionals
- Supports for patients

### Role and responsibilities of professionals in the consent process

#### Junior doctors

Of the junior doctors, 38 (67%) were trainees in the medical speciality and 19 (33%) in surgical rotations. Of these, 55 (96%) had been tasked by a senior member of their medical team to discuss and obtain consent, verbal or written from a patient for a planned intervention. The survey questions asked about surgery or procedure or treatments. From here on, this is simplified to ‘intervention’.

Most junior doctors (26, 46%) reported taking consent several times a week. The majority (63%) of consent episodes occurred in an inpatient setting (ward), and 39% of the patients consented had a planned intervention within 24 h. All of the junior doctors, both interns and SHOs, reported that they were not supervised by a more senior doctor during consent taking. Seventeen per cent of the doctors (10) had seen or attended the planned intervention before obtaining consent from patients. When breaking this down between interns (*n* = 3) and SHOs (*n* = 6), the number doubled for the SHOs.

#### Nursing professionals

Amongst the respondents, the majority (49, 27%) work as Clinical Nurse Specialist (CNS), followed by 25% (45) as Clinical Nurse Manager II (CNMII) and 18% (33) were professionals nurses most of whom (85, 49%) work in an outpatient setting. Within their current role, 47% (52) of the nursing professionals reported that they are responsible for obtaining verbal or implied consent directly from the patients, while 56% (62) are responsible for helping patients access the necessary information to make informed decisions. Nurses (24, 22%) are also responsible for securing patients’ signatures on consent forms.

#### Comparison between junior doctors and nursing professionals

The survey’s findings indicate that junior doctors and nursing professionals are both responsible for obtaining patient consent, as a significant number of respondents were involved in the consent process, but their roles differ. Among the nurses who responded, the majority supported the patients to access the information they needed to enable the patient to make an informed decision, while the majority of doctors were responsible for holding consent conversations in relation to planned interventions with patients and obtaining written consent from patients.

### Information and knowledge

#### Junior doctors

Of the junior doctors, at the time that consent was discussed with the patient, most felt they had enough information and knowledge to explain the purpose of the planned surgery or procedure to the patient (49, 91%); the benefits of the planned surgery or procedure (49, 91%); alternative interventions available to them including no intervention (36, 67%); and risks and/or possible complications of the planned intervention (47, 87%). Seventy percent of junior doctors (38) did not provide written information to the patient about the planned intervention. The results are presented in Table 1.

**Table 1:** Junior doctors’ responses in relation to the questions about their knowledge and provision of information

Question		Responses	Count & percentage of total responses received
I feel that at the time I discussed consent with the patient I had enough information and knowledge about the planned surgery or procedure to do the following effectively:	Explain the purpose of the planned surgery or procedure to the patient	Not stated No Yes	3 5 (9%) 49 (91%)
	Describe the planned surgery or procedure to the patient	Not stated No Yes	3 9 (17%) 45 (83%)
	Inform the patient about the benefits of the planned surgery or procedure	Not stated No Yes	3 5 (9%) 49 (91%)
	Inform the patient of the alternative treatments available to them including no surgery/procedure	Not stated No Yes	3 18 (33%) 36 (67%)
	Inform the patient about the risks and/or possible complications of the planned surgery or procedure	Not stated No Yes	3 7 (13%) 47 (87%)
	Answer any questions that the patient might ask	Not stated No Yes	3 20 (37%) 34 (63%)
I provided the patient with written information about the planned surgery/procedure		Not stated No Sometimes Yes	3 38 (70%) 11 (20%) 5 (10%)

#### Nursing professionals

Forty-five per cent of the nurse respondents (49) felt adequately knowledgeable about the planned intervention before undertaking consent discussion with the patient and 40% of nursing professionals (44) reported always being able to provide the patient with written information about their planned intervention.

#### Comparison between junior doctors and nursing professionals

Most of the junior doctors felt that during the consent discussion with the patient, they had enough information and knowledge to explain the purpose of the planned intervention, whilst less of the nursing professionals felt they had sufficient knowledge about the planned intervention before undertaking the consent discussion with the patient.

### Education and training

#### Junior doctors

Just under one-third of junior doctors (17) felt that they received adequate information and training to undertake consent discussions with patients competently. Over one-fifth (12 of junior doctors felt they had adequate opportunities to observe senior team members undertaking consent discussions with patients, 44% (23) felt confident that they could undertake effective consent discussions with patients independently, and 37% of doctors (19) felt confident that their verbal and written consenting practices met with current legal requirements.

In the event that junior doctors did not feel that they had sufficient knowledge about a planned intervention to undertake the consent discussion with a patient, they would seek information and support from different sources including when a patient was assessed as not having capacity to make this particular decision (Table 2).

**Table 2:** Junior doctors’ responses in relation to the questions about education and training

Question	Responses	Count and percentage of total responses received
I receive adequate information and training to undertake consent discussions with patients competently	Not stated No Not sure Yes	5 24 (46%) 11 (21%) 17 (33%)
I receive adequate support and supervision from my senior team members to undertake consent discussions with patients competently	Not stated No Not sure Yes	5 33 (63%) 5 (10%) 14 (27%)
I have adequate opportunities to observe my senior team members undertaking consent discussions with patients	Not stated No Not sure Yes	5 37 (71%) 3 (6%) 12 (23%)
I am confident that I can undertake effective consent discussions with patients independently	Not stated No Not sure Yes	5 7 (14%) 22 (42%) 23 (44%)
I am confident that my verbal and written consenting practices meet with current legal requirements	Not stated No Not sure Yes	5 8 (15%) 25 (48%) 19 (37%)
In the event I did not feel adequately familiar with a planned surgery/procedure to undertake the consent discussion with a patient, I would	Not stated Inform my supervisor that I was not comfortable to take consent Ask a senior colleague for help with the consent discussion Source information on the planned surgery/procedure and continue with the task Continue and hope the patient does not ask questions I cannot answer	6 19 (30%) 13 (20%) 29 (45%) 3 (5%)
In the event I required additional information about a planned surgery/procedure, I would source it from the following	Not stated A senior colleague/consultant An agreed (by my Team) on line resource Any on-line resource	5 25 (30%) 19 (22%) 41 (48%)
In the event I felt the patient did not have the capacity to consent to a planned surgery/procedure, I would:	Not stated Inform my supervisor of the situation Ask my supervisor/senior colleague for help with the consent discussion Obtain written consent from the patient’s family/carer Engage with my consultant, team, and patient’s family in making a consent decision that was in the best interest of the patient	5 25 (40%) 23 (37%) 9 (15%) 5 (8%)

#### Nursing professionals

Of the nursing professional respondents, 58% (63) felt that since joining the hospital they had not received information on good consent practices, and 48% (*n* = 52) reported that they needed training on consent processes and practices. Further training needs expressed included in relation to understanding patients’ capacity (30%), the legal aspects of consent (29%), informed consent best practices (23%), and the provision of patient information material (6%).

### Supports for professionals

#### Junior doctors

The majority (32, 56%) of junior doctors reported that there were no patient information materials (PIMs) or fact sheets related to the planned intervention that could be provided to patients. Most (35, 61%) of the doctors reported that they felt that they had adequate time to discuss everything they needed to with patients. The hospital’s standard consent form was used by 93% of junior doctors (53) to record the discussion and the patient’s consent decision. This enabled the creation of a complete record of the discussion and the patient’s consent decision according to most junior doctors (35, 61%). Most doctors (42, 74%) felt confident with how they had established the patient’s consent.

#### Nursing professionals

Thirty-two (29%) of the nursing professionals encountered patients who changed their minds after having initially given consent to the proposed intervention. Thirty respondents described how they dealt with this situation, e.g. asking the consultant for help (Table 3).

**Table 3:** Content analysis results for dealing with those who declined to give their consent

Theme	Count and percentage (out of 55 strategies)
Referred to consultant/doctors	14 (25%)
Provided education/more information/answering questions	14 (25%)
Acknowledged fears/listened to concerns/reassured patient	12 (22%)
Cancelled procedure/treatment/offered to reschedule	7 (13%)
Other options/alternative treatments were discussed	4 (7%)
Involved families/relatives	2 (4%)
Involved nursing staff	2 (4%)

Fifty-one (47%) nursing respondents reported experiencing a time when a patient was assessed as not having the capacity to consent to an intervention. Following on from this question, 47 respondents described their reported ways of dealing with this situation including involving family members (56%), consulting medical team (20%), and involving legal team (21%).

### Supports for patients

#### Junior doctors

Junior doctors (37, 69%) felt that patients would have adequate information to make an informed decision about agreeing (consenting) or not to their planned intervention following a discussion with them. Junior doctors (28, 58%) also reported that they felt that the patient had enough time to consent or not to the planned intervention.

#### Nursing professionals

Most nurses (94, 91%) ensure that patients consent to the care that they provided to them. Content analysis of the 129 response from 92 nursing professionals to the follow-up question ‘If yes, how do you do this’ indicates that (57, 42%) of nurses obtain verbal confirmation by undertaking further discussion and education including the provision of PIMs (50, 37%), by confirmation and checking that the consent form has been signed (6, 4%), and asking the patient to explain the procedure to ensure the patient understands (5, 4%).

### Results of free text survey responses

#### Barriers to good consent practices

Thirty-nine (68%) junior doctors described 56 barriers to consent practices. For doctors, the highest ranking barriers related to (i) the appropriateness of the clinician undertaking consent conversations (doctors with less experience and knowledge) (23%), (ii) patient informed and given PIMs (21%), and (iii) time to understake consent conversations (18%). Eighty-two nursing respondents described 121 barriers of which the highest ranking barriers included (i) time for consent conversations (18%), (ii) language barriers (18%), and nurses feeling ill equipped through the lack of education to undertake consent conversations (11%). Table 4 presents the combined thematic analysis of the barriers to good consent processes and practices. It can be seen here that adequate time and the provision of good understandable information for patients are the highest ranked barriers.

**Table 4:** Thematic analysis for barriers to good consent practices

Theme	Count and percentage (out of 177 barriers)
Time for consent conversations ‘Time to properly consent patients and allow them an opportunity to think over the conversation’	34 (19%)
Patient centred and patient information materials (PIMs) ‘Linked paper resources to give to patients’	25 (14%)
Language barriers ‘Language barriers for either the patient or the clinician, e.g. accent, lack of available interpreter’	24 (14%)
Clinician experience/education in relation to consent ‘Interns have more limited knowledge in what is involved in the procedure being consented for. SHOs/Regs are not often on the wards to consent for procedures’	22 (12%)
Appropriate clinician having consent conversation	13 (7%)
Staffing levels for consent conversations	12 (7%)
Appropriate environment to have consent conversations	10 (6%)
Communication barriers	10 (5%)
Consent form	6 (3%)
Other (e.g. PPPGs, simplification of the process, information on risks and benefits, workload, lack of resources)	21 (12%)

### Suggestions for improvements

Thirty-six junior doctors gave 47 suggestions for improvement the highest ranking being better information for patients (38%), followed by education for professionals in relation to consent practices (17%) and improvements to the hospital consent form (15%). Ninety-one nurses gave 116 suggestions for improvements the highest ranking being more education and training (22%), provision of more PIMs (15%), and more time for consent conversations (12%). Table 5 presents the combined thematic analysis of the suggestions to improve consent processes and practices. It can be seen here that the development and provision of PIMs for patients and more education for clinical staff along with creating more time for consent conversations were the highest ranking suggestions for improvement.

**Table 5:** Thematic analysis of suggestions to improve consent practices

Theme	Count and percentage (out of total 163 suggestions)
More information to be provided to patients (PIMs) including on risks and benefits and in different languages ‘Patient information leaflets relevant to procedure… to this hospital. Able to provide to patient’	52 (32%)
More education for clinical staff on all aspects of consent ‘Update my knowledge’	36 (22%)
More time to have consent conversations	17 (10%)
Development of PPPGs and standardised procedures for consent processes, simplification of the consent process, having designated consent officers to help with standardisation	15 (9%)
Provision of an appropriate environment in which to have consent conversations in private	13 (8%)
Improve the consent form	7 (4%)
Ensure most appropriate clinician engaging in consent conversations	6 (4%)
Provision of electronic supports for consent	6 (4%)
Other (e.g. involving mix of professionals in consent processes, increasing staffing levels)	11 (7%)

## Discussion

The process of informed consent is central in the planning and provision of safe, effective person-centred healthcare as it encompasses HCPs and patients communicating about, and together deciding on and agreeing to medical interventions. This survey-based study looked at the experiences, attitudes, and perceived needs of junior doctors and nursing professionals in relation to the informed consent process in clinical practice at an acute hospital.

Most junior doctor respondents were confident in describing the purpose and benefits of the intervention to the patient. This corresponds to our finding in Kovic et al.’s [26], where the majority of patients were satisfied that the decision they made to consent was informed. They felt that their clinician explained the treatment and provided them with information about the benefits. Less doctors (37%) reported being confident that their written consenting practices meet with current legal requirements, aligning with other findings [13–15]. Similar less than half of nurses reported feeling adequately knowledgeable about the planned intervention before discussing consent with patients and identified the need for more training on consent processes and practices.

Junior doctors and nurses need further education and training on consent. Many of the doctors reported that they have never been observed by senior team members while obtaining consent or have never witnessed the planned intervention beforehand, again in line with other studies [12, 13]. Heaney et al. [12] argue whether interns should be involved in the consent process; however, in a qualitative study by Wood et al. [7], consultants acknowledged that junior doctors may lack experience in obtaining valid informed consent, but neither should they be completely excluded from this role, as it provides them with opportunities to learn and develop their skills in relation to the informed consent process [7].

Both the medical and nursing professions have identified training, education, knowledge, and experience as barriers to ensuring good, informed consent practices. These aspects also resonate with other studies where the lack of time and unfamiliarity with procedures affect the quality and amount of information conveyed to patients and the overall appropriateness of the clinician undertaking consenting [7, 11]. There is no standardised or consistent approach to teaching informed consent skills [32], but simulation training has proved beneficial [33]. Nurses also require further education on consent both within institutional settings and through academic curricula. Axson et al. [27] found nurses felt undergraduate education did not prepare them to actively engage in informed consent processes, and Strini et al. [34] found that nurses with less work experience consider their training sufficient, while those with more experience feel it is inadequate, demonstrating increased awareness of educational needs.

Kovic et al. [26] found that the public’s perspective of voluntary consent included having other options explained, being provided with adequate time to discuss options with their family and carers, being reassured that one can decide otherwise at any stage without negative consequences, the opportunity to talk to other medical teams in the same specialities, and having the opportunity to seek a second opinion in another hospital. Results of this study have shown that voluntariness can be challenging since the period between when junior doctors obtain consent and patients undergo a procedure is frequently rather short to allow patients to process all the information which may potentially impair the voluntariness of consent or lead to a rushed decision. Junior doctors in our study have voiced this sentiment, with 42% believing that patients do not have enough time to process information for informed consent.

Both doctors and nurses identified time as a barrier in our study as in other studies [7, 11, 19, 20, 26, 27]. The lack of time also affects the quality and amount of information conveyed to patients and the overall appropriateness of clinician consenting [7], which affects voluntariness as decisions may be rushed.

Doctors and nurses in this study believe it is important to improve the quality of the PIMs provided to patients, to make PIMs more easily available for HCPs to provide to patients and also to improve the way and approach that information is provided to patients regarding their procedures and treatment. In addition, doctors expressed a need to improve the consent form, making it specific to the procedure and written in simple language so that it is easy to understand and accessible. HCPs also need to have effective communication skills to be able to talk to patients and discuss any risks and benefits with them [26].

Junior doctors and nurses reported engaging the patient’s family in obtaining signed consent. The misunderstanding around when it is appropriate to contact a family member or ‘next of kin’ (NOK) as well as the changes implemented by the ADMCA need to be addressed in junior doctors’ and nurses’ training to facilitate good consent practices [35]. Additional training may include the use of health information, making reasonable adjustments such as longer and/or multiple appointments, offering the option of a support person to attend, using plainer language, involving the individual in discussions, and communicating effectively.

### Recommendations for improvement

Overall, this study has revealed that there are many system aspects involved in obtaining valid informed consent. Studying the perceptions and attitudes of junior doctors and nursing professionals from the same hospital has allowed us to identify the following recommendations for improvement:

- To develop PIMs that are co-designed by HCPs and patients, with details on procedures including risks and benefits of the procedure and other treatment options, to ensure the information addresses HCP and patients’ needs. One of the suggestions, for example, arising from the Kovic et al. [26] study was that patient and members of the public want to know more about how their lives will change following interventions; this aspect is often not covered in current PIMs.
- To develop and deliver education and training resources for HCPs in relation to consent practices, SDM and ADM. There are several opportunities available to increase HCPs understanding and facilitate them gaining experience, including making recommendations to undergraduate and postgraduate academic curricula, providing more opportunities to observe senior team members engaging in consent conversations, providing professionals with written PPPGs which also aligns with other studies [7], and exploring the introduction of different mediums for education, e.g. simulation training [36].
- To map out the instances where consent conversations currently happen in the patient’s journey and co-design between HCPs and patients’ new processes and pathways to build in additional time for consent conversations to take place at additional and/or alternative moments throughout the patient journey that patients value. For example, Wood et al. [7] found that early discussions are important to patients, e.g. during the patient’s pre-operative hospital visit [7], and Kovic et al. [26] found that patients also want subsequent opportunities to ask questions once they have processed this initial information.
- To review the current paper-based hospital consent form to better support HCPs and patients to engage in good consent practices in relation to interventions. To enable the integration of the paper-based form with the Electronic Health Record (EHR).
- To introduce electronic supports for the new co-designed consent conversations and PIMs. This would enable iterative updating of PIMs and interactive communication between HCPs and patients before, during, and after any intervention. Electronic supports would further enable ongoing dialogue between HCPs and patients and allow patients to process information at their own pace throughout their healthcare journey [36]. Electronic supports would allow opportunities for HCPs to discuss treatment options remotely with patients, allow patients to discuss options with their family/carers, and allow patients time to form questions. Electronic supports would also allow for maintaining a record of consent discussions that could be make available to those involved in the patient’s healthcare journey. The introduction of electronic tools and moving toward digital consent has been shown to increase the quality of consent conversations and SDM [36, 37].
- To provide more appropriate environments where confidentiality and privacy can be better safeguarded, without distractions, to engage in consent conversations and SDM.

### Limitations

This study took place at one hospital site, and the generalisability of its findings may be questionable; however, although the response rate was low, it is within the expected response rate and was similar to other studies conducted in Ireland. Survey-based studies are by their nature limited in what they can discover about the reasons behind participants’ choice of answers. We did include a free text response section to elicit comments on the current barriers to good consent practices and suggestions for improvement. Further focus groups or observation-based studies would add to the information gathered here. This study does however take into account the perspectives of both junior doctors and nursing professionals, thus providing a more holistic view of current and desired consent processes and practices.

## Conclusions

The process of informed consent is central in the planning and provision of safe, effective person-centred healthcare as it encompasses healthcare professionals and patients communicating about, and together deciding on and agreeing to medical interventions. This survey-based study looked at the experiences, attitudes, and perceived needs of junior doctors and nursing professionals in relation to the informed consent process in clinical practice at an acute hospital and informed the development of recommendations for improvement.

## Data Availability

All data generated or analysed during this study are included in this published article (and its supplementary information files).

## Appendix 1

Junior doctors’ questionnaire.

## Appendix 2

Nurses’ questionnaire

## Abbreviations

ADMCA: Assisted Decision-Making (Capacity) Act
ADON: Assistant Director of Nursing
ANP: Advanced Nurse Practitioner
cANP: Candidate Advanced Nurse Practitioner
CNM: Clinical Nurse Manager
CNS: Clinical Nurse Specialist
HCP: Healthcare Professional
HSE: Health Service Executive
NCHD: Non-Consultant Hospital Doctors
PIL: Participant Information Leaflets
PIM: Patient Information Materials
PPPG: Policies, Procedures, Protocols and Guidelines
SDM: Shared Decision-Making
SHO: Senior House Officers
